# Effect of Eccentricity Difference on the Mechanical Response of Microfluidics-Derived Hollow Silica Microspheres during Nanoindentation

**DOI:** 10.3390/mi15010109

**Published:** 2024-01-08

**Authors:** Hao Wu, Juzheng Chen, Tianyi Jiang, Wenlong Wu, Ming Li, Shanguo Zhang, Ziyong Li, Haitao Ye, Mengya Zhu, Jingzhuo Zhou, Yang Lu, Hongyuan Jiang

**Affiliations:** 1 School of Mechatronics Engineering, Harbin Institute of Technology, Harbin 150001, China; 2 Department of Mechanical Engineering, City University of Hong Kong, Kowloon, Hong Kong SAR 999077, China; 3 Nano-Manufacturing Laboratory (NML), City University of Hong Kong Shenzhen Research Institute, Shenzhen 518057, China; 4 Department of Mechanical Engineering, The University of Hong Kong, Pokfulam Road, Hong Kong SAR 999077, China

**Keywords:** microfluidics, double-emulsion droplet, hollow microsphere, nanoindentation, FEM

## Abstract

Hollow microspheres as the filler material of syntactic foams have been adopted in extensive practical applications, where the physical parameters and their homogeneity have been proven to be critical factors during the design process, especially for high-specification scenarios. Based on double-emulsion droplet templates, hollow microspheres derived from microfluidics-enabled soft manufacturing have been validated to possess well-controlled morphology and composition with a much narrower size distribution and fewer defects compared to traditional production methods. However, for more stringent requirements, the innate density difference between the core–shell solution of the double-emulsion droplet template shall result in the wall thickness heterogeneity of the hollow microsphere, which will lead to unfavorable mechanical performance deviations. To clarify the specific mechanical response of microfluidics-derived hollow silica microspheres with varying eccentricities, a hybrid method combining experimental nanoindentation and a finite element method (FEM) simulation was proposed. The difference in eccentricity can determine the specific mechanical response of hollow microspheres during nanoindentation, including crack initiation and the evolution process, detailed fracture modes, load-bearing capacity, and energy dissipation capability, which should shed light on the necessity of optimizing the concentricity of double-emulsion droplets to improve the wall thickness homogeneity of hollow microspheres for better mechanical performance.

## 1. Introduction

Hollow microspheres are lightweight microparticles with spherically symmetric morphology, which have been adopted in a myriad of practical applications ranging from construction [1,2,3,4], transportation [5,6,7], chemical engineering [8,9,10,11], pharmaceutical research [12,13,14,15], and so forth. Due to their outstanding advantages in buoyancy performance [16,17,18], energy absorption [19,20,21], thermal/acoustic insulation [22,23,24], etc., ceramic hollow microspheres have shown great superiority as filler materials to regulate the performance of syntactic foam, which is a kind of classical composite material extensively used under various circumstances [25,26,27,28]. It is well known that the geometrical characteristics, such as wall thickness, shell diameter, as well as their ratio, have played a decisive role in determining the overall physical properties of hollow microspheres, including the effective density, specific surface, isostatic pressure resistance, etc. [29,30,31]. Moreover, the overall geometric homogeneity is also of vital importance for the actual usage based on the fact of the massive quantity involved, which fundamentally requires a narrower size distribution and a lower percentage of defects for the hollow microsphere yield. This inevitably raises the threshold of the manufacturing process, undoubtedly posing challenges for traditional manufacturing methods in terms of adding excessive sorting and screening processes as well as concurrently increasing costs.

To date, typical industrial hollow microspheres, represented by the fly ash cenospheres, which are the by-product of coal combustion in thermal power plants, have been systematically analyzed and proven to possess significant geometric heterogeneity [32,33,34]. The actual dimension of individual hollow microspheres varies and the range can reach up to several times the nominal size, which has a negative influence on the preliminary design of syntactic foam, especially for high-standard applications. This large geometric inhomogeneity is deeply rooted in the manufacturing process; i.e., the preparation of the powdery raw material by milling shall ineluctably result in the heterogeneity of granular dimensions, and the ingredient content of the blowing agent cannot be precisely controlled. In addition, the melting kinetics of the discrepant powdery compounds in identical high-temperature smelting conditions change synchronously and, thus, maintain the differentiation [35,36]. Other common manufacturing methods, such as spray technology, fluidized bed, suspension process, etc., all have their shortcomings with respect to the geometric homogeneity of hollow microspheres. In terms of spray technology, there is an apparent contradiction between the pursuit of prescribed droplet dimensions and the minimization of geometric heterogeneity, since external forces, such as electrical, pneumatic, and mechanical forces, are generally introduced to regulate droplet dimensions at the cost of increasing the difficulty of reducing the droplet polydispersity. Additionally, the competition between mass transport velocity and potential droplet supersaturation usually reduces the yield. For the fluidized bed and suspension methods, the geometric homogeneity of the as-prepared hollow microspheres is largely determined by two critical factors: one is the degree of the monodispersity of the solid particles as sacrificial templates, and the other is the subsequent surface coating effect. The fluidized bed approach is inferior for handling small particles down to 50 μm in real industrial production because the particles are prone to agglomeration, and the subsequent coating methods, such as sputtering, laser ablation, CVD deposition, spraying, etc., all require multiple precautions to guarantee the coating uniformity, which can remarkably improve the complexity of the manufacturing process [37,38,39]. The suspension technique is capable of realizing precise coating on smaller particles through the precipitation and agglomeration of colloidal particles or the alternating the layer-by-layer interfacial reaction at the nanoscale. However, this process is relatively complicated, since in each coating step, not only the molar ratio of raw materials should be strictly regulated to prevent potential flocculation or bridging phenomena but also the non-absorbed particles should be separated in a timely manner [40,41,42,43], thereby significantly increasing the costs of time and economy.

In recent years, microfluidics-enabled soft manufacturing has emerged as a robust technological means to fabricate liquid templates with well-controlled composition and morphology, which has already innovated the fabrication approach in various fields, encompassing chemistry, biology, physics, and engineering [44,45,46,47,48,49,50,51,52]. The easily prepared double-emulsion droplets with a core–shell structure possess a series of advantages, including controllable compositions, adjustable dimensions, and a higher degree of structural homogeneity, which have already been exploited to fabricate hollow microspheres via the sintering of ceramic nanoparticles inside the shell solution [53,54,55]. Compared with traditional industrial manufacturing methods, it is promising to downsize the shell size discrepancy to several microns with a shell regulation range of hundreds of microns, but a slight deficiency lies in the innate density difference between the core/shell solutions, which may cause an eccentric problem if no additional regulation measures are taken, further leading to the uneven distribution of the wall thickness for the hollow microsphere.

Herein, in order to investigate the influence of wall thickness inhomogeneity on the mechanical performance of microfluidics-derived hollow silica microspheres, a hybrid method combining a nanoindentation experiment and FEM simulation was performed. The FEM model was firstly benchmarked against the existing experimental data and, thus, established to reveal the nominal strain distribution under different indentation displacements as well as the disparate fracture modes of hollow silica microspheres with two different eccentricities, which should shed light on the importance of improving the overall geometric homogeneity, including both the inner wall thickness and the outer shell diameter, of the hollow microspheres.

## 2. Materials and Methods

### 2.1. Preparation of the Microfluidic Device

**Figure 1:**
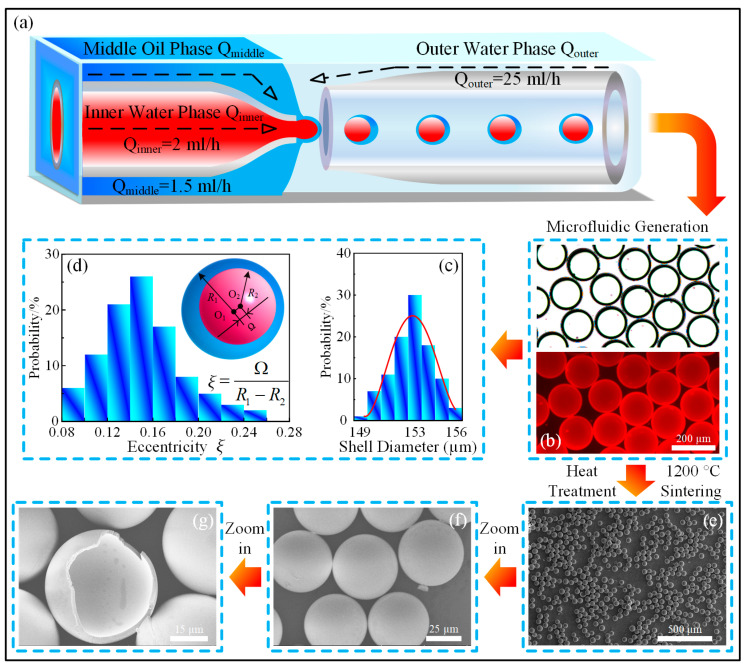
Schematic illustration of the microfluidics-based hollow silica microsphere fabrication process. (**a**) Schematic diagram of the microfluidic system. (**b**) The optical and fluorescence images of the as-prepared W1/O/W2 double-emulsion droplets; the scale bar is 200 µm. (**c**) Statistical analysis of the shell diameter of the as-prepared W1/O/W2 double-emulsion droplets. (**d**) Statistical analysis of the eccentricity of the as-prepared W1/O/W2 double-emulsion droplets. (**e**,**f**) SEM images of the as-prepared hollow silica microspheres based on microfluidics. The scale bars are 500 µm and 25 µm, respectively. (**g**) SEM image of an artificially crushed hollow silica microsphere. The scale bar is 15 µm.

As shown in Figure 1a, the microfluidic device for W1/O/W2 double-emulsion droplet generation was assembled by sequentially inserting and fixing two cylindrical glass capillary tubes at each end of a square glass tube. The tips of the two cylindrical glass capillary tubes were forged into different sizes, i.e., 40 µm and 260 µm, with the smaller cylindrical glass capillary tube used as the channel for the inner water phase Q_inner_, while the larger one served as the collection tube for the W1/O/W2 double-emulsion droplets. The interstices between the two cylindrical glass capillary tubes and the square glass tube were the inlets of the middle oil phase Q_middle_ and the outer water phase Q_outer_, respectively. The area through which the middle oil phase flowed was treated with trimethoxy (octadecyl) silane to make it hydrophobic.

### 2.2. Materials

Both the inner and outer water phases were 5 wt% poly (vinyl alcohol) solution, while the middle oil phase was the mixture of photoinitiator 2-hydroxy-2-methylpropiophenone, photosensitive monomer ethoxylate trimethylolpropane triacrylate, fluorescent red pigment, and silica nanoparticle-loaded xylene solution, in which the poly (vinyl alcohol), trimethoxy (octadecyl) silane, 2-hydroxy-2-methylpropiophenone, and ethoxylated trimethylolpropane triacrylate were provided by Sigma-Aldrich. The silica nanoparticle-loaded xylene solution was purchased from Jingcai Chemical Co., Guangzhou, China, and the red pigment was bought from Aladdin, Shanghai, China. The deionized water was prepared using the Millipore Milli-Q system, Saint Louis, MO, USA.

### 2.3. Preparation of the Hollow Silica Microspheres

The hollow silica microspheres were prepared from the generated W1/O/W2 double-emulsion droplets by high-temperature sintering at 1200 °C. During this heat treatment process, all the organic substances were thermally pyrolyzed, while the remaining silica nanoparticles were gradually subjected to precipitation, aggregation, and calcination processes. This high-temperature smelting process made the microsphere shell void-free and more compact, as demonstrated in Figure 1.

### 2.4. Characterization

The optical and corresponding fluorescence images of the as-prepared double-emulsion droplets were obtained with an optical microscope system (BX53, Olympus, Tokyo, Japan) coupled to a high-speed CCD camera (DP27, Olympus, Tokyo, Japan). The SEM pictures were captured with a scanning electron microscope (SU8010, Hitachi, Tokyo, Japan), and some hollow microspheres were intentionally crushed to show the internal morphology. The nanoindentation tests were carried out with a commercial nanoindenter (G200, Agilent Technologies, Santa Clara, CA, USA) equipped with a 100 μm flat indenter.

## 3. Results and Discussion

### 3.1. Microfluidic Fabrication and Characterization of the Hollow Microspheres

As shown in Figure 1a–d, the W1/O/W2 double-emulsion droplets as soft templates for hollow microsphere fabrication could be easily prepared by the microfluidic device with a rational three-phase flow rate combination. It could be found from the statistical analysis results that the shell diameters of the as-prepared droplets presented a sort of small-range distribution, which was the merit of this microfluidics-enabled soft manufacturing approach. However, the eccentricity ξ, which was defined by the quotient of the deviation distance Ω between the two centers O_1_, O_2_, and the radius difference (R_1_–R_2_) of the core/shell droplets, showed a random distribution pattern and was expected to deteriorate further due to the innate solution density difference or any potential perturbation.

**Figure 2:**
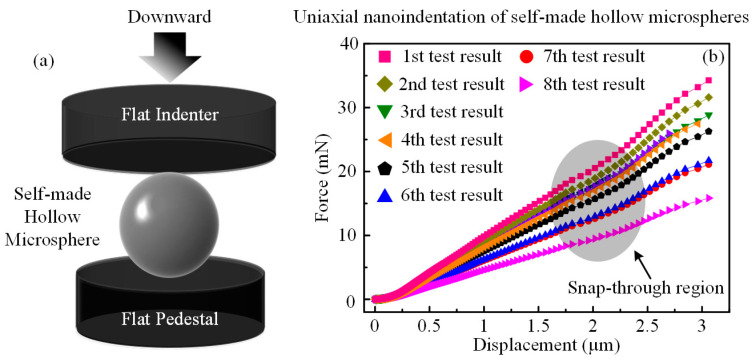
(**a**) Schematic representation of the uniaxial nanoindentation test setup equipped with a flat indenter. (**b**) Nanoindentation results of hollow silica microspheres derived from the microfluidic W1/O/W2 double-emulsion droplets.

This geometric disadvantage of the soft template could be inherited by the hollow microspheres after heat treatment in the form of wall thickness heterogeneity, as depicted in Figure 1e–g. As a result, the mechanical properties of the hollow microspheres should be discretized in a plausible range. Thus, in order to verify this inference, nanoindentation tests, as schematically illustrated in Figure 2a, were performed using a 100 μm flat indenter, and the loading velocity was set to 3 nm/s. Since the average shell diameter of silica hollow microspheres was about 45 μm, the much larger indenter could effectively meet the axial centering requirement, thereby minimizing the influence of artificial factors. The experimental force-displacement results of the nanoindentation tests were listed in Figure 2b, where all the curves possessed a snap-through region around 2 μm, as highlighted by the gray oval shadow. This indicated the occurrence of the shell buckling phenomenon, which could release the accumulated stress during incipient compression. However, it should be noted that although all the hollow microspheres have undergone the same indentation displacement of 3 μm, their mechanical responses could vary to a certain extent, with the maximum value reaching up to more than twice the minimum counterpart. Based on the approximation hypothesis that all hollow microspheres shared basically identical outer shell diameters, this difference should be attributed to the inner geometric inhomogeneity, i.e., the uneven distribution of the wall thickness. In order to verify this deduction, two FEM models were constructed using the commercial software ABAQUS^TM^ SIMULIA Suite 2017 and were further calibrated to reproduce the trend of the force-displacement curves of the tested hollow microspheres during nanoindentation down to 3 μm. Thus, the compression process, in particular the specific fracture modes of two types of hollow microspheres with different eccentricities, could be revealed, thereby clarifying the evolution of the strain distribution as the hollow microspheres were deformed at further indentation displacements.

### 3.2. Simulation-Based Mechanical Response Analysis of Hollow Silica Microspheres with Varying Eccentricities

The two typical hollow microspheres were modeled with an identical mean wall thickness of 2.5 μm, outer diameters of 45 μm, and varying eccentricities of 0, i.e., perfectly concentric, and 0.6, i.e., comparatively eccentric, respectively. The loading configuration of the eccentric hollow microsphere was set to arrange the loading direction parallel to the connection between the thinnest and thickest shell walls. Due to identical structure, boundary conditions, and applied forces during the compression process, only a quarter of the hollow microsphere was modeled under symmetry boundary conditions to simplify the model. This resulted in a spatial displacement degree of freedom and two spatial rotational degrees of freedom being zero at each of the two orthogonal symmetric planes. The corresponding settings are listed in Table 1.

**Figure 3:**
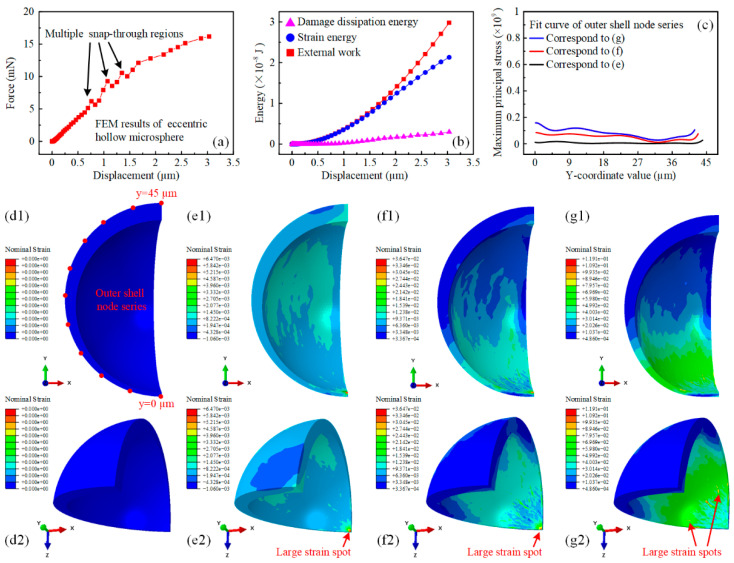
Finite element simulation results of eccentric hollow microsphere nanoindentation with prescribed displacement. (**a**) Force-displacement behavior of the eccentric hollow microsphere during uniaxial indentation simulation. (**b**) The variation of external energy input, strain energy, and damage dissipation energy of the eccentric hollow microsphere during uniaxial indentation simulation. (**c**) The numerical fit curve of the maximum principal stress distributed in the outer shell of the eccentric hollow microsphere with respect to the y coordinates at varying displacements. (**d**–**g**) The nominal strain distribution of the eccentric hollow microsphere during uniaxial indentation simulation. (**d1**,**d2**) The nominal strain distribution contour of the eccentric hollow microsphere from two different perspectives in the initial configuration. (**e1**,**e2**) The nominal strain distribution contour of the eccentric hollow microsphere from two different perspectives at a displacement of 1 µm. (**f1**,**f2**) The nominal strain distribution contour of the eccentric hollow microsphere from two different perspectives at a displacement of 2 µm. (**g1**,**g2**) The nominal strain distribution contour of the eccentric hollow microsphere from two different perspectives at a displacement of 3 µm.

After the iterative run and calibration of the FEM model, the simulation results of the eccentric hollow microspheres with 3 μm feed were shown in Figure 3. The thinnest part of the hollow microsphere was mechanically weak and prone to deform due to its comparative eccentric configuration and the gradual increase in external work. The force-displacement curve of the eccentric hollow microsphere displayed several snap-through regions at smaller compression feeds. The load capacity, which refers to the magnitude of the force concerning the nanoindentation displacement, decreased accordingly.

Figure 3b shows that the external work was dissipated only as strain energy before the snap-through phenomenon occurred. Afterward, the damage dissipation energy began to increase slowly but accounted for only a small portion of the external work input. This indicated that the thin-walled region could withstand relatively large strain rather than rapid fracture. The distribution profile of the nominal strain in Figure 3d–g explicitly demonstrates the energy conversion process. It was observed that the total nominal strain of the eccentric hollow microsphere was proportional to the nanoindentation displacement. Additionally, the strain magnitude of the thin-walled region was much higher than that of the thick-walled counterpart. Furthermore, the extent of large strain areas in the thin-walled region gradually increased from the bottom tip to encompass the entire lower hemisphere, as illustrated in Figure 3e–g. Simultaneously, the maximum strain spot shifted from the bottom to the lower part, as indicated by the red arrows, demonstrating the occurrence of buckling phenomena in the previously constrained thin-walled region in direct contact with the pedestal. To enable quantitative comparison, Figure 3c shows the numerical fit curves of the maximum principal stress distributed in the outer shell of the eccentric hollow microsphere at different displacements. The distribution of the maximum principal stress was consistent with the nominal strain. At a displacement of 1 µm, the maximum principal stress was induced at both ends of the hollow microsphere, while the remaining regions remained essentially stress-free. For further displacements, significant increases in the maximum principal stress were observed in the expanding areas of the thin-walled region with small y-coordinate values. However, the stress level was lower due to the mechanically inferior thin-walled geometry.

**Figure 4:**
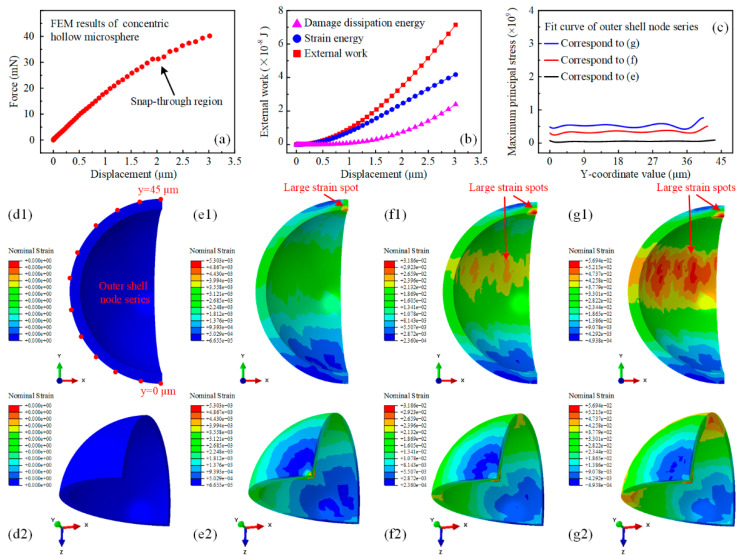
Finite element simulation results of concentric hollow microsphere nanoindentation with prescribed displacement. (**a**) Force-displacement behavior of the concentric hollow microsphere during uniaxial indentation simulation. (**b**) The variation of external energy input, strain energy, and damage dissipation energy of the concentric hollow microsphere during uniaxial indentation simulation. (**c**) The numerical fit curve of the maximum principal stress distributed in the outer shell of the concentric hollow microsphere with respect to the y coordinates at varying displacements. (**d**–**g**) The nominal strain distribution of the concentric hollow microsphere during uniaxial indentation simulation. (**d1**,**d2**) The nominal strain distribution contour of the concentric hollow microsphere from two different perspectives in the initial configuration. (**e1**,**e2**) The nominal strain distribution contour of the concentric hollow microsphere from two different perspectives at a displacement of 1 µm. (**f1**,**f2**) The nominal strain distribution contour of the eccentric hollow microsphere from two different perspectives at a displacement of 2 µm. (**g1**,**g2**) The nominal strain distribution contour of the eccentric hollow microsphere from two different perspectives at a displacement of 3 µm.

The concentric hollow microsphere did not have an obvious mechanically weak region due to its symmetric geometry, making it mechanically stronger with perfect wall thickness homogeneity. Figure 4a shows that the snap-through region occurred at approximately 2 μm, consistent with the experimental results. The corresponding force magnitude was approximately 2.7 times that of the eccentric hollow microsphere. Meanwhile, the concentric hollow microsphere was able to sustain much larger external work. Although dissipated in identical forms, namely, the strain energy and damage dissipation energy, their ratio varied accordingly. The damage dissipation energy started to increase significantly corresponding to the snap-through region and accounted for half of the strain energy at the displacement of 3 μm, reaching about eight times that of the eccentric hollow microsphere. Furthermore, the homogeneity of the wall thickness has altered the deformation mode of the hollow microsphere. This was verified by examining the nominal strain distribution in Figure 4d–g. At an indentation displacement of 1 μm, the nominal strain distribution did not exhibit any excessive regional concentration features, except for a single large strain spot located directly at the inner shell under the indenter. The strain in the upper middle part of the shell was relatively larger. This could be attributed to the bulging deformation of the concentric hollow microsphere caused by compression. As a result, the external work was dissipated in the form of strain energy. At an indentation displacement of 2 μm, another large strain spot appeared, as indicated by the red arrow. The nominal strain contour presented an approximately symmetric distribution with this spot as the center of symmetry with respect to the indentation direction. It should be noted that the nominal strain distribution in the lower hemisphere of the concentric hollow microsphere was comparatively smaller, which differed from the results of the eccentric counterpart. For an indentation displacement of 3 μm, the large strain spot in the upper hemisphere continued to strengthen in both scope and magnitude, as shown in Figure 4g. This spot would serve as the vulnerable region for further indentation. To quantitatively compare the maximum principal stress, the fitting curves of the outer shell node series at varying indentation displacements are displayed in Figure 4c. Furthermore, the lower section of the concentric hollow microsphere did not exhibit a significant stress concentration. This resulted in a considerable enhancement in stress magnitude, highlighting the importance of improving the homogeneity of wall thickness in hollow microspheres for superior mechanical performance.

### 3.3. Simulation-Based Prediction of Damage Evolution Behavior of Hollow Silica Microspheres with Varying Eccentricities

**Figure 5:**
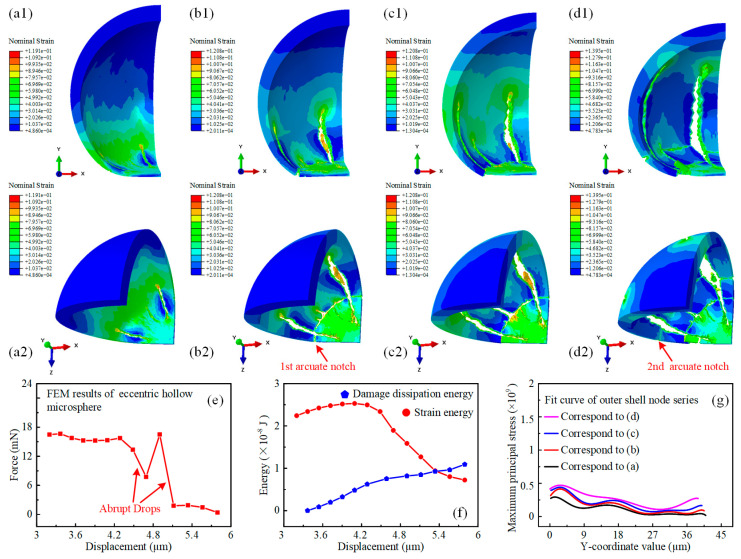
Finite element simulation results of eccentric hollow microsphere nanoindentation with further displacements. (**a**–**d**) The nominal strain distribution of the eccentric hollow microsphere during the simulation of further uniaxial indentation. (**a1**,**a2**) The nominal strain distribution contour of the eccentric hollow microsphere from two different perspectives at the displacement of 4 µm. (**b1**,**b2**) The nominal strain distribution contour of the eccentric hollow microsphere from two different perspectives at the displacement of 4.5 µm. (**c1**,**c2**) The nominal strain distribution contour of the eccentric hollow microsphere from two different perspectives at the displacement of 5 µm. (**d1**,**d2**) The nominal strain distribution contour of the eccentric hollow microsphere from two different perspectives at the displacement of 5.5 µm. (**e**) The force-displacement behavior of the eccentric hollow microsphere during simulation of further uniaxial indentation. (**f**) The variation of strain energy and damage dissipation energy of the eccentric hollow microsphere during the simulation of further uniaxial indentation. (**g**) The numerical fit curve of the maximum principal stress distributed in the outer shell of the eccentric hollow microsphere with respect to the y coordinates at varying displacements.

Although no experimental data were available for reference, it was still possible to predict the subsequent failure and damage evolution behavior of the hollow microspheres through simulation. The respective fracture modes were also found to be closely correlated with their eccentricities, as shown in Figure 5a–d. The evolution of the nominal strain distribution revealed the fracture development process of the eccentric hollow microsphere. The strain spots in the lower thin-walled hemisphere developed into cracks along the longitudinal direction, as shown in Figure 5a. These cracks then extended upwards, tearing the hemisphere and resulting in the formation of an arcuate notch along the latitudinal direction, as indicated by the red arrow in Figure 5b. This represented the disconnection of the most fragile thin-walled region. Therefore, the load-bearing section of the eccentric hollow microsphere was reduced, and the newly formed arcuate notch acted as the new bottom in direct contact with the platen. Subsequently, a new longitudinal crack originated from the first arcuate notch, and the previous cracks continued to grow toward the upper thick-walled hemisphere. Subsequently, the shell of the eccentric hollow microsphere underwent another fracture, and the second arcuate notch occurred in the thin-walled region, as highlighted by the red arrow in Figure 5d. The damage evolution behavior of the eccentric hollow microsphere exhibited a repeated partial fracture mode starting from the thin-walled region, which corresponded to the abrupt drops in the force-displacement curve in Figure 5e. The sudden decrease in the load-bearing capacity of the eccentric hollow microsphere was caused by the formation of latitudinal cracks, specifically the two arcuate notches, in the thin-walled region. 

The strain energy decreased during the repeated shell collapse procedure and was released by the initiation and propagation of cracks, particularly the formation of transverse arcuate notches. Meanwhile, the damage dissipation energy increased proportionally to the indentation displacement as more cracks developed. It was an interesting phenomenon worth noting. Figure 5g shows the numerical fit curves of the maximum principal stress distribution for the shell node series, used for quantitative analysis and validation. The stress concentration region was located at the thin-walled hemisphere, and the overall stress level increased with the indentation displacement. The stress magnitude in the thick-walled part began to increase distinctly only after the emergence of the second arcuate crack, which was consistent with the nominal strain distribution.

**Figure 6:**
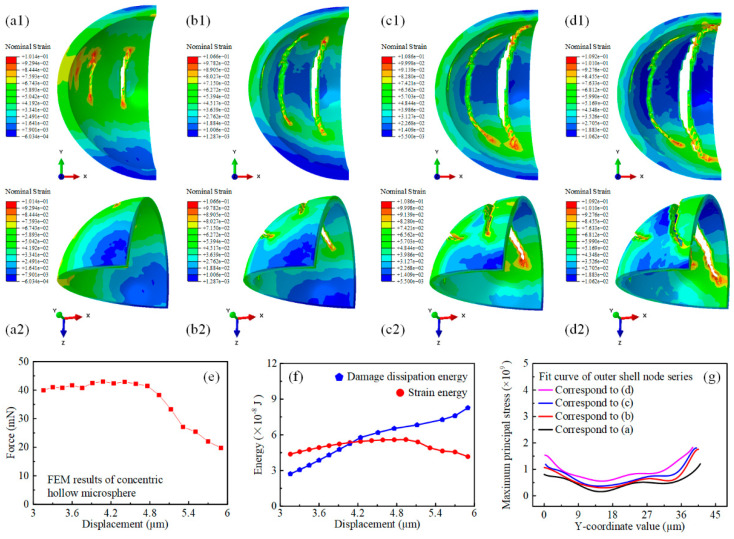
Finite element simulation results of concentric hollow microsphere nanoindentation with further displacements. (**a**–**d**) The nominal strain distribution of the concentric hollow microsphere during the simulation of further uniaxial indentation. (**a1**,**a2**) The nominal strain distribution contour of the concentric hollow microsphere from two different perspectives at the displacement of 4 µm. (**b1**,**b2**) The nominal strain distribution contour of the concentric hollow microsphere from two different perspectives at the displacement of 4.5 µm. (**c1**,**c2**) The nominal strain distribution contour of the concentric hollow microsphere from two different perspectives at the displacement of 5 µm. (**d1**,**d2**) The nominal strain distribution contour of the concentric hollow microsphere from two different perspectives at the displacement of 6 µm. (**e**) The force-displacement behavior of the concentric hollow microsphere during simulation of further uniaxial indentation. (**f**) The variation of strain energy and damage dissipation energy of the concentric hollow microsphere during the simulation of further uniaxial indentation. (**g**) The numerical fit curve of the maximum principal stress distributed in the outer shell of the concentric hollow microsphere with respect to the y coordinates at varying displacements.

Likewise, the damage evolution process of the concentric hollow microsphere was also initiated from the large strain spots, as depicted in Figure 6. The longitudinal cracks formed due to the perfect homogeneity of the wall thickness. These cracks extended synchronously along both ends and gradually lengthened with respect to the indentation displacement. The large nominal strain was concentrated at the crack tips, causing the hollow microsphere to be torn up and split into several lobes. Each lobe was sandwiched between the indenter and the platen. The longitudinal segmentation of the concentric hollow microsphere differed significantly from the layer-by-layer latitudinal collapse of the eccentric hollow microsphere. Despite maintaining a similar strain magnitude to the eccentric counterpart, the concentric hollow microsphere exhibited an improved load capacity of over two-fold, and the force magnitude did not decrease until the cracks approached the top and bottom of the shell, as illustrated in Figure 6e. This trend was similar to the variation tendency of the strain energy. The strain energy was not significantly released until the damage dissipation energy reached a certain level, as shown in Figure 6f. Figure 6g displays the fitting curves for the quantitative analysis of the maximum principal stress on the outer shell. Unlike the eccentric hollow microsphere, the stress magnitude at both ends of the concentric hollow microsphere was at the same level, which was significantly larger than that in the middle part due to the initiation and development of cracks in the middle region. Therefore, all the curves presented a basin-like trend, with the maximum value exceeding twice that of the eccentric counterpart. Therefore, the simulation results suggested that the eccentricity, or wall thickness homogeneity, of the hollow microsphere was a crucial factor in determining its specific mechanical responses, including deformation modality, crack evolution paths, and fracture modes. In addition, optimizing the concentricity of the double-emulsion droplets during the preparation process of hollow microspheres using a microfluidics-enabled soft manufacturing method could effectively improve the mechanical performance of the microspheres in terms of the force/stress magnitude and energy dissipation capability.

## 4. Conclusions

Herein, a hybrid method combining experimental nanoindentation and FEM simulation was used to characterize the mechanical response of microfluidics-derived hollow silica microspheres with varying eccentricities. The eccentric hollow microsphere exhibited a repeated partial fracture mode during the indentation process through the formation of transverse arcuate notches. Meanwhile, longitudinal cracks gradually developed and propagated upwards toward the thick-walled region due to the indentation displacement. The load-bearing capacity and energy dissipation capability of the eccentric hollow microsphere were comparatively lower due to the presence of mechanically weak thin-walled regions. Due to the homogeneity of the wall thickness, only longitudinal cracks formed in the concentric hollow microsphere during the indentation process. These cracks continued to expand synchronously along both ends and gradually lengthened, tending to split the hemisphere into several lobes. The concentric hollow microsphere exhibited significantly improved mechanical performance compared to the eccentric counterparts, and the load capacity and energy dissipation capability increased by more than two-fold. Therefore, it was both reasonable and necessary to enhance the concentricity of the double-emulsion droplets in the microfluidics-based fabrication process of hollow microspheres. This improvement in homogeneity of the wall thickness could provide a more stable geometric foundation for achieving better mechanical performance, thereby meeting the higher requirements in practical applications.

## Figures and Tables

**Table 1:** Settings of the ABAQUS^TM^ model.

Modeled Hollow Microsphere	Physical Parameters of Microsphere
Part description: 3D deformable	Young’s Modulus: 76 GPa
Materials behavior: brittle cracking	Poisson’s ratio: 0.22
Section type: Solid, homogeneous	Simulated Diameter: 45 μm
Element type: Linear tetrahedron C3D10	**Modeled Indenter and Pedestal**
Through thickness elements: 4	Part description: 3D analytical rigid

## Data Availability

The data that support the findings of this study are available from the corresponding author upon reasonable request.
